# The Role of Monoclonal Antibodies in the Management of Leukemia

**DOI:** 10.3390/ph3103258

**Published:** 2010-10-18

**Authors:** Ali Al-Ameri, Mohamad Cherry, Aref Al-Kali, Alessandra Ferrajoli

**Affiliations:** 1 Department of Leukemia, the University of Texas MD Anderson Cancer Center, Houston, TX 77005, USA; 2 Department of Internal Medicine, Hematology Oncology section, Oklahoma University Health Sciences Center, 700 N.E. 13th Street, Oklahoma City, OK 74103, USA

**Keywords:** monoclonal Abs, leukemia, CLL, AML, ALL

## Abstract

This article will review the monoclonal antibodies more commonly used in leukemias. In the last three decades, scientists have made considerable progress understanding the structure and the functions of various surface antigens, such as CD20, CD33. The introduction of rituximab, an anti CD20 monoclonal antibody, had a great impact in the treatment of lymphoproliferative disorders. Gemtuzumab, an anti CD 33 conjugated monoclonal antibody has activity in acute mylegenous leukemia (AML). As this field is undergoing a rapid growth, the years will see an increasing use of monoclonal antibodies in hematological malignancies.

## 1. Introduction

In 1900, speaking of monoclonal antibodies (MAbs), Paul Ehrlich proposed that “immunizations such as these which are of great theoretic interest may come to be available for clinical application attacking epithelium new formations, particularly carcinoma by means of specific anti-epithelial sera” [1]. Erlich’s dream came true with the first report of the manufacturing of MAb in 1975 by Kohler and Milstein [2,3]. However, the effects of such products have been realized only in recent years, particularly in the treatment of leukemia and non-Hodgkin lymphoma. The use of rituximab, particularly in combination with various chemotherapy and radiotherapy regimes, has significantly improved the outcome of several types of lymphoma [4,5]. In addition, rituximab is being used for the treatment of other hematological disorders ranging from malignancies such as chronic lymphocytic leukemia (CLL) to autoimmune disorders such as immune and thrombotic thrombocytopenic purpura and rheumatoid arthritis [6]. In this review, we describe some of the MAbs with activity in leukemias (Table 1).

**Table 1:** Monclonal ABs in lymphoproliferative disorders.

MAb	Target antigen	Isotype	Indication
XmAB 5574	CD19	Humanized and FC enhanced, mouse IgG1	Preclinical development
Rituximab	CD20	Humanized mouse IgG1	CLL, Follicular and diffuse large B cell NHL
Ofatumumab	CD20	Fully humanized IgG1	Phase I-II CLL Follicular B cell NHL
Ocrelizumab	CD20	Humanized IgG1	Phase II Follicular B cell NHL
Veltuzumab	CD22	Humanized and enhanced IgG1	Phase I Follicular B cell NHL
Epratuzumab	CD22	Humanized IgG1	Phase I-II Follicular B cell NHL and CLL
Inotuzumab ozogamicin	CD22	Humanized IgG4-N-acetyl-y1-calicheamiein	Preclinical development
BI22 ha22	CD22	Murine, IgG1 (pseudomonas exotoxin)	Phase I-II HCL and B cell CLL
Lumiliximab	CD23	Cynomolgus macaque-human IgG1	Phase I-II-III CLL
Alemtuzumub	CD52	Humanized rat IgG2b	CLL, T-PLL

## 2. Anti-CD20 Antibodies

The past three decades have seen considerable progress in our understanding of the structure and function of the CD20 molecule and the development of engineered anti-CD20 MAbs. CD20 MAbs mechanism of action include: complement-dependent cytotoxicity (CDC), programmed cell death, and Fc: FCR-dependent interactions, with passive immunization being a potential fourth mechanism [6]. Although it is widely accepted that Fc-Fc gamma receptor (FcgR) interactions are critical in follicular lymphoma, the role of the complement-dependent pathway and the mechanism of programmed cell death in other lymphoproliferative disorders are still disputed [6]. Rituximab is a chimeric murine-derived MAb that targets the CD20 antigen from the anti-CD20 antibody on the human constant region. Complement-mediated cell lysis is induced by rituximab. CD20 is a good target because of its high expression and the proximity of the AB binding epitope to the plasma membrane. After binding by MAb CD20 is redistributed in the insoluble lipid rafts, which appears to be important for clustering the MAb, leading to C1q and then CDC binding [7]. In regard to the role played by complement, an *in vivo* study demonstrated that complement levels decreased after rituximab infusion and that restoring the complement led to a resumption of the activity of rituximab in CDC [7]. In vitro, anti-CD20 MAb was shown to induce CDC in follicular lymphoma cells, and this induction was regulated by the complement inhibitors CD55 and CD59 [8]. 

Weng and Levy [9] tested this concept in patients, and also found that rituximab caused CDC in follicular lymphoma (FL) cells. CD45, CD55, and CD59 expression were assessed in pretreatment FL cells from 29 patients treated with rituximab. Of these patients, eight had a complete response (CR), 11 had a partial response (PR), and 10 either no response or a minimal one. These researchers then determined the expression of CD20, CD46, CD55, and CD59 by flowcytometry and found that pretreatment expression of these markers by lymphoma cells did not predict clinical outcome after rituximab treatment [9]. Another study found that too great a tumor burden, such as bulky lymphomas, exhausted body effector mechanisms such as natural killer (NK) cell-mediated immunity, which substantially decreased the effect of rituximab in cell killing and suggested that in cases of a large tumor burden, the complement component becomes disadvantageous to the efficacy of rituximab [10]. 

Williams *et al.* [11] conducted a pilot study in patients with CLL aiming to study the shaving mechanism of rituximab, in which the CD20 complex is stripped from B cells by monocytes. The hypothesis of that study was that frequent lower doses of rituximab might reduce shaving and produce adequate targeting and clearance of CD20^+^ B lymphocytes from the circulation. Results suggested that when the threshold rituximab dose is exceeded, additional doses will not clear further CD20^+^ cells. Finally, Racila *et al.* [12] hypothesized that complement may play a role in the clinical response to rituximab and other MAb-based cancer therapies. They found that polymorphisms in the C1qA gene affect the clinical response and the duration of response to rituximab therapy in patients with follicular lymphoma [12].

Rituximab has activity against CLL cells despite the low antigen density of CD20 on the surface of CLL cells [9]. This activity is mediated through several mechanisms: antibody-mediated cellular cytotoxicity, complement-dependent cytotoxicity, and apoptosis induced through the activation of caspase-3, which sensitizes cells to proapoptotic stimuli [10,11,12].

Rituximab was first tested in CLL as a single agent in several phase I studies in which administered doses varied widely, from 10 to 500 mg/m^2^. There was little agreement about the most effective dose for CLL. Thus, the weekly regimen of 375 mg/m^2^ was selected empirically [13,14,15,16,17]. Single-agent rituximab has a low response rate, but its activity in combination makes it one of the most important discoveries so far for the management of CLL. 

Hainsworth [18] administered rituximab 375 mg/m^2^ weekly for four weeks to 44 patients with previously untreated CLL/SLL who had at least one indication for treatment. The overall response (OR) rate was 58%, with a CR rate of 9%; the 2-year progression-free survival (PFS) was 49%. When rituximab was given (375 mg/m^2^ weekly for eight weeks) as a front-line agent to 31 patients with Rai stage 0–2 CLL, no standard indications for treatment and high β_2_ microglobulin levels, the OR rate was 90% and the CR rate was 19%. Two dose-escalation studies were conducted in an attempt to improve response rates in patients with recurrent CLL with higher doses and more intense schedules evaluated by O’Brien and Byrd. The response rates were higher in this setting (up to 75%) than with standard doses [19]. Currently, Rituximab is not routinely used as a single agent in front line therapy for CLL patients as the combination with Fludarabine with or without cyclophosphamide is clearly superior. The fact that the higher doses produced a higher response in the recurrent setting raises the question whether higher doses should be the new standard of care.

### 2.1. Chemo Immunotherapy for CLL

Several groups evaluated rituximab in combination with different chemotherapy agents active against CLL. All of these studies indicated that combination chemo-immunotherapy had superior efficacy. The Cancer and Leukemia Group B treated 104 previously untreated patients with an indication for therapy consisted of fludarabine 25 mg/m^2^ on days 1–5 of a 28-day cycle for a total of six cycles with or without rituximab 375 mg/m^2^ on days one and four for the first cycle, followed by weekly rituximab at the same dose for four weeks. The results showed that the fludarabine plus rituximab arm had a higher over all response rates of 90%, compared with 77% for the fludarabine-only arm. The CR rate was 47% in the fludarabine plus rituximab arm and 28% in the fludarabine-only arm [20,23]. 

Keating and colleagues designed a regimen of fludarabine, cyclophosphamide, and rituximab (FCR; fludarabine 25 mg/m^2^ and cyclophosphamide 250 mg/m^2^ daily for 3 days plus rituximab 375 mg/m^2^ on day 1 of cycle 1 and 500 mg/m^2^ on day 1 of cycles 2–6). This regimen was given to 177 patients with relapsed or refractory CLL [20]. Therapy with FCR was generally well tolerated and yielded an OR rate of 73%; CR was achieved by 25% of patients, of whom 32% also had a complete molecular response. Tam *et al.* [22] reported the results with the same FCR regimen in 300 patients with previously untreated CLL and found an OR rate of 95% and a CR rate of 72%. The 6-year overall and disease-free survival rates were 77% and 51%, respectively, with a median time to progression of 80 months [23]. A retrospective multivariate analysis of patients receiving fludarabine-based therapy at MDACC revealed FCR therapy to be the strongest independent determinant of survival [24]. The German CLL Group compared FCR and fludarabine plus cyclophosphamide in a large phase III randomized trial (CLL8). In this study, Eight hundred seventeen treatment naive patients with CLL were randomized 1:1 to FCR or Fc alone. In a recent update, FCR induced a higher OR rate (95% *vs.* 88%) and more CRs (52 % *vs.* 27%; *P* < 0.0001) than Fc, as well as an improved PFS of 52 months *vs.* 33 months for the Fc group (*P* < 0.001). Most significantly, patients in the FCR arm had an improved OS compared with those in the Fc arm. At 38 months, patients in the FCR arm had an OS of 84 % *vs.* 79% (*P* = 0.01) in the Fc arm. The greatest benefit from the FCR regimen was seen in the patients with Binet A and B (69% of all patients). The statistically significant differences in OS were only seen in the patients with Binet A and B. The CLL8 is the first phase III study to demonstrate a statistically significant improvement in clinical benefit with FCR compared to Fc in a randomized trial. The German CLL Study Group now considers FCR to be the standard of care for front-line management of advanced CLL [25].

### 2.2. Rituximab in Acute Lymphoid Leukemia (ALL)

The management of ALL has changed significantly during the past 20 years, stem cell transplant, tyrosine kinase inhibitors, and rituximab have been incorporated into ALL treatment. Although the CD markers are used as diagnostic tools, their prognostic implications are not clear. In rituximab studies, CD20 expression was evaluated, and a cut point of 20% by mean fluorescence intensity was chosen [48]. Studies concluded that absolute CD20 expression and the increase in mean fluorescence intensity were both independently associated with decreased event-free survival irrespective of other prognostic factors [49]. Thomas *et al.* [49] evaluated the significance of CD20 expression in 253 adult and adolescent patients with de novo precursor B-cell ALL treated by combination chemotherapy regimens of VAD with or without cyclophosphamide or hyper-CVAD. Forty-seven percent of patients were CD20 positive. Current results suggest that the use of rituximab in combination with cyclophosphamide is advantageous in younger patients with ALL [50].

### 2.3. Rituximab in Burkitt’s Lymphoma

Burkitt’s lymphoma (BL) is an aggressive B-cell non-Hodgkin lymphoma characterized by deregulation of the c-MYC gene and a high degree of proliferation of malignant cells. Thomas *et al.* reported their experience in patients with *de novo* BL and ALL treated with the combination of hyper-CVAD and rituximab. The results obtained compared favorably with the historical controls indicating that the addition of rituximab may improve outcome [50].

Dunleavy *et al.* included standard-dose rituximab in other previously established regimens for BL, such as a modified dosing approach with cyclophosphamide, vincristine, doxorubicin, and high-dose methotrexate (CODOX-M) alternating with ifosfamide, etoposide, and high-dose cytarabine (IVAC) and a dose-adjusted approach with etoposide, prednisone, vincristine, cyclophosphamide, and doxorubicin. They found encouraging preliminary results with such combinations. Several studies have recently shown that the use of rituximab and intensive chemotherapy for HIV-related BL is feasible and can result in outcomes similar to those in the HIV-negative population, particularly in the setting of effective highly active antiretroviral therapy [51].

Gregory *et al.* recently [57] reported the results of 12 patients with Burkitt’s lymphoma enrolled in the first of two stage investigator initiated trials. Low risk (LR) patients were defined as normal LDH, stage I/II, ECOG <2, and no mass >10 cm. All other pts were high risk (HR). LR pts received 3 CODOX-M cycles, while HR pts received four alternating cycles of CODOX-M and IVAC. Liposomal doxorubicin (40 mg/m^2^) was used in lieu of doxorubicin (day 1 CODOX-M) and high dose rituximab (500 mg/m^2^) was added days 0 + 8 of CODOX-M and days 0 + 6 of IVAC. Results: 3/12 patients had HIV+ disease, and 9/12 had HR disease. The overall response rate after two cycles was 100% (11/11) with a complete remission rate (CRR) of 82% (9/11). The 2-year PFS and OS rates are 73%, while the cause-specific survival is 82%. One patient died in CR from unknown causes, while two patients died due to progressive disease. The authors concluded that the incorporation of rituximab and liposomal doxorubicin to CODOX-M/IVAC for BL appears safe and efficient and is numerically improving the overall survival compared to historic control data (73% *versus* <65%).

## 3. Ofatumumab

Ofatumumab is a humanized anti-CD20 monoclonal antibody. Ofatumumab has a slow off rate, high CDC activity, and binds to an epitope [26] in the smaller loop of the CD20 antigen. The clinical efficacy and safety of single-agent ofatumumab has been reported in two phase I-II trials of relapsed/refractory CLL and FL, and phase III trials are ongoing. Ofatumumab was effective in a group of patients with fludarabine- and alemtuzumab-resistant CLL, who are known to have a poor prognosis. Combination chemotherapy with ofatumumab is currently being investigated [27].

Recently, Wierda *et al.* reported the results from an international, randomized, phase II trial with two doses of ofatumumab combined with fludarabine and cyclophosphamide in previously untreated patients with CLL; 61 previously untreated patients with active CLL were randomized to receive either 500 mg or 1,000 mg of ofatumumab on day 1, combined with daily fludarabine (25 mg/m^2^) and cyclophosphamide (250 mg/m^2^) on days 1–3, every four weeks for a total of six courses. In both groups, the first dose of ofatumumab was 300 mg. The complete response rate was 32% for those who received 500 mg of ofatumumab compared with 50% for those who received 1,000 mg of ofatumumab; the ORR was 77% and 73%, respectively. The median PFS was not reached at the median follow-up of 8 months. No grade 3–4 infusion related reactions on the day of ofatumumab infusion were reported. Investigators concluded that this combination regimen is highly active in previously untreated CLL patients.

## 4. Other Anti CD20 MAbs

Ocrelizumab, veltuzumab and GA101. These new MAbs are being engineered for potential benefits over the first-generation rituximab; modifications to second-generation include a humanized IgG1 MAb to reduce immunogenicity but with an unmodified Fc region, and the third-generation MAbs are humanized and have an engineered Fc region designed to improve therapeutic performance by adapting their effector functions [26].

### 4.1. Anti-CD52 Antibodies

*Alemtuzumab:* Alemtuzumab (Campath-1H) is a MAb that recognizes the CD52 antigen, a 21- to 28-kd, heavily glycosylated, membrane-anchored glycoprotein that is expressed on B, T, and NK cells, on macrophages, and invariably expressed by CLL cells. In contrast, granulocytes, platelets, erythrocytes, and hematopoietic stem cells lack CD52 expression. Cross-linking CD52 on B-cell lines resulted in growth inhibition followed by induction of apoptosis [29,30].

Phase I studies of alemtuzumab conducted in patients with relapsed CLL established a dose of 30 mg intravenously three times per week for 4–12 weeks, given at 3 mg on day 1, 10 mg on day 2, 30 mg on day 3, and 30 mg three times per week thereafter. Phase II studies have shown that alemtuzumab is effective in patients with relapsed/refractory CLL. Similar efficacy, in CLL but markedly reduced infusion related events were achieved when alemtuzumab was administered subcutaneously [30].

Alemtuzumab is indicated as therapy for patients with B-CLL refractory to fludarabine [31]. In a pivotal trial, 93 patients with fludarabine-refractory CLL received alemtuzumab, treatment rendered an OR rate of 33%, a CR rate of 2%, a median time to progression of 9.5 months, and a median OS of 16 months. Alemtuzumab was effective at eliminating CLL cells from the peripheral blood and bone marrow however, was less effective in patients with adenopathy >5 cm in diameter and those with poor performance status [32]. Patients with the del 17p13 cytogenetic abnormality, which confers an adverse prognosis in CLL, have also been shown to respond to alemtuzumab [33]. 

The CAM307 trial was an open-label, multicenter, randomized trial that compared alemtuzumab relative to chlorambucil in untreated patients with Rai stage I–IV CLL requiring treatment. A total of 297 patients were adaptively randomized by Rai stage, age, performance status, sex, and maximum lymph node size. Based on an intention-to-treat analysis, alemtuzumab yielded a higher OR rate (83% *vs.* 55%), CR rate (22% *vs*. 2%), MRD eradication rate (31% *vs.* 0%), time to alternative treatment (23.3 *vs.* 14.7 months), and PFS [34].

Moreton *et al.* highlighted the importance of achieving a response in a study designed to administer alemtuzumab to 91 patients for a median of nine weeks. Of these patients, 36% had a CR, including 18 (20%) who achieved minimal residual disease (MRD)-negative status by flow cytometry. The most important conclusion in this study was that patients with CR and no evidence of MRD by flow cytometry had a survival advantage [29].

Consolidation therapy with alemtuzumab in patients with MRD positivity was assessed in 23 patients with CLL who had responded to initial chemotherapy with fludarabine alone or combined with cyclophosphamide and were randomized to observation or alemtuzumab (30 mg intravenously three times per week for 12 weeks [35]. Of the 21 assessable patients, 11 were randomized to receive alemtuzumab, and severe infections were observed in seven of them, which included life-threatening pulmonary aspergillosis (n = 1), grade 3 cytomegalovirus reactivation (n = 4), pulmonary tuberculosis (n = 1), and herpes zoster (n = 1). This high incidence of severe infection led to the premature closing of the trial. Two of 11 patients in the alemtuzumab arm converted from PR to CR, and five of six assessable patients had MRD eradication in peripheral blood, whereas all patients in the observation arm remained MRD-positive [36].

It is worth noting that rituximab as a single agent or in combination can induce MRD and this was demonstrated in a phase II randomized trial comparing rituximab with or without interferon in symptomatic patients with advanced indolent lymphoma [58]. Overall, 73/128 patients (58%) were responders (CR or PR), and among those patients who attained a clinical CR and were tested for MRD by PCR, 54% achieved sustained molecular remissions for up to five years (rituximab in combination with fludarabine and cyclophosphamide whether used as a frontline regimen or in relapsed disease in patients with CLL, has demonstrated high response rates, with 21/75 patients achieving MRD negativity [21,24].

Alemtuzumab has also shown activity in combination with fludarabine. (OR rate 83% and CR rate 30%). This was evaluated in The CAM314 [59] (phase III open-label, randomized study) in patients with progressive chronic lymphocytic leukaemia (CLL) that was recently reported by Engert *et al.* The study involving 335 patients with relapsed or refractory (one prior therapy) Rai stage I-IV CLL investigated whether treatment with alemtuzumab/fludarabine (FluCAM) was more beneficial than treatment with fludarabine alone. With a median follow-up of 17 months, the (FluCAM) arm improved the ORR from 68% to 85%, CR rate from 16% to 30%, and PFS from 18.1 months to 23.8 months (P = 0.01). Those patients with Rai III-IV disease showed an improvement in PFS from 12.1 to 26.1 months with (FluCAM) (P = 0.003). Overall survival, did not reach significance. Adverse events were similar in both arms, including cytopenias and infections. Additionally, as well as when combined the combination of alemtuzumab with fludarabine, cyclophosphamide, and rituximab has also showed some benefit (CFAR regimen; OR rate 65% and CR rate 24%) in patients with relapsed CLL.A trial evaluating the activity of CFAR in the frontline setting is under way [34]. Toxicity, in particular myelosuppression and infectious complications, are common with these combination regimens, and prophylaxis in mandatory.

*Combined rituximab and alemtuzumab*: The rationale for combining alemtuzumab and rituximab is based on targeting different antigens on the surface of B-cells with agents that have different mechanisms of action. Alemtuzumab is effective in depleting CLL cells that are circulating and in the bone marrow, whereas rituximab has activity against CLL cells in the lymph nodes, and both antibodies have favorable toxicity profiles.

Faderl *et al.* [35] reported on the activity and safety of rituximab in combination with alemtuzumab in 48 patients with relapsed or refractory lymphoid malignancies, including 32 with CLL, nine with CLL/prolymphocytic leukemia, one with PLL, four with mantle cell lymphoma, and two with Richter transformation. Treatment consisted of rituximab at a dose of 375 mg/m^2^ weekly for four weeks and alemtuzumab given at the loading-dose schedule of 3 mg, 10 mg, and 30 mg on three consecutive days during the first week of treatment, followed by a dose of 30 mg on days 3 and 5 of weeks 2–4. Although patients could receive a second 28-day cycle, depending on their response and ability to tolerate the first cycle, only seven (15%) patients did so. The OR rate (52%) was higher than that previously reported for alemtuzumab given as single agent. Responses were observed in 25 (52%) patients, including four (8%) with CR, two (4%) with nodular PR, and 19 (40%) with PR. The OR rate among the 32 patients with CLL was 63%, which included two patients who achieved CR and 16 patients who achieved PR. After a median follow-up of 6.5 months, the median time to progression was six months [35].

Finally, alemtuzumab increases the vulnerability of CLL patients to opportunistic infections, including fatal bacterial, viral, fungal, and protozoal infection, as well as reactivation of cytomegalovirus (CMV). Appropriate antibacterial and antiviral prophylaxis should be instituted when Alemtuzumab is used. In the package insert, infections can occur in 43 to 74% of patients, CMV viremia in 55% and CMV infection in 6%–16%. CMV viremia should be measured by quantitative PCR every 2 to 3 weeks during treatment. Valganciclovir prophylaxis is used by some centers if viremia is present while others use it only if the viral load is rising.

### 4.2. Anti-CD33 Antibodies

CD33 is a 67-kDa transmembrane cell-surface glycoprotein that is specific for myeloid cells whose expression is down-regulated with maturation of the myeloid lineage and thus is expressed at low levels on peripheral granulocytes and tissue macrophages. CD33 is expressed in approximately 90% of patients with AML, with expression defined by the presence of the antigen on more than 20% of leukemic blasts but not on normal CD34, pluripotent hematopoietic stem cells, or in nonhematopoietic tissues. *In vitro* studies have shown that the CD33/anti-CD33 complex is internalized by target cells [37].

Gemtuzumab ozogamicin is a humanized IgG4 anti-CD33 MAb (hP67.6) conjugated to calicheamicin, a potent intercalating agent. In the acidic environment of the lysosome, the derivative dissociates from the antibody and binds inside the minor groove of DNA, causing double-stranded DNA breaks. Lentivirus-mediated gene transfer to manipulate CD33 expression in myeloid cell lines that normally lack or have very low levels of CD33 has proved the quantitative relationship between CD33 expression levels and the *in vitro* response to gemtuzumab ozogamicin. Furthermore, AML blasts from patients whose disease responded to the drug had significantly higher mean CD33 levels and lower P-glycoprotein activity than did nonresponders, and CD33 expression and P-glycoprotein activity had an inverse correlation [38].

The maximum tolerated dose of gemtuzumab was found in phase I dose escalation studies to be 9 mg/m^2^, and this was verified by CD33 receptor site saturation data as the appropriate dose for phase II studies. A 14-day dosing interval was chosen based on the half-life of the antibody [38]. Larson *et al*. [39] found that some patients with relapsed or refractory AML in their study exhibited clearance of bone marrow blast cells with incomplete platelet recovery. In patients with relapsed AML a phase II study of gemtuzumab was associated with an overall response rate of 26% and a median disease-free survival of 5.2 months. One percent of patients developed severe liver failure caused by sinusoidal injury with features similar to those of veno-occlusive disease of the liver; the risk of this adverse event was especially high in patients that subsequently underwent hematopoietic stem cell transplantation [41]. Single-agent gemtuzumab ozogamicin in older patients with newly diagnosed AML has shown OR rates (complete remission plus incomplete platelet recovery) seldom exceeding 30%–35% [42,43]. Gemtuzumab ozogamicin appears to be poorly tolerated in patients older than 75 years, and a dosage reduction has been proposed for these patients [43].

It should be noted that gemtuzumab ozogamicin has recently been withdrawn from the market in the USA. An action that was taken by the manufacturing company at the request of the US Food and Drug Administration. Gemtuzumab ozogamicin was approved to treat patients ages 60 years and older with recurrent AML who were not considered candidates for other chemotherapy. A confirmatory, post approval clinical trial was begun by Wyeth (now Pfizer) in 2004. The trial was designed to determine whether adding gemtuzumab ozogamicin to standard chemotherapy demonstrated an improvement in clinical benefit (survival time) to AML patients. The trial was stopped early when no improvement in clinical benefit was observed, and after a greater number of deaths occurred in the group of patients who received gemtuzumab ozogamicin compared with those receiving chemotherapy alone.

*Lintuzumab* is a humanized anti-CD33 MAb derived from the murine M195 antibody. Lintuzumab is similar to its murine parent M195 with respect to specificity, immunoreactivity, and internalization properties. However, lintuzumab has higher avidity than mouse M195, and, because the murine constant regions were replaced with human immunoglobulin sequences, it has the ability to mediate leukemia cell killing [44].

In phase I trials, immunotherapy with lintuzumab was well tolerated and produced objective responses in seven of 17 patients. Among 50 patients with relapsed or refractory AML, one PR and two CRs were achieved in patients with low marrow blast infiltration (5%–30%). In a phase III trial of lintuzumab (12 mg/m^2^) combined with induction chemotherapy (mitoxantrone 8 mg/m^2^, etoposide 80 mg/m^2^, and cytarabine 1 mg/m^2^, given daily for six days) was compared with chemotherapy alone in 191 adults with first relapsed or primary refractory AML. The response rate with the combination regimen was 36%, compared with 28% in patients who received chemotherapy alone; the overall median survival was 156 days in both arms of the study and no differences in chemotherapy-related adverse effects were observed with the addition of the lintuzumab to standard chemotherapy [45,46,47]. Thus, this MAb can be incorporated into treatment strategies that can achieve minimal disease states. In a phase II study, patients with newly diagnosed APL in clinical complete remission immediately after all-trans retinoic acid and/or chemotherapy were treated with lintuzumab twice weekly for 3 weeks [47]. Among the 31 patients treated in first remission, 28 had minimal residual disease detectable by reverse transcriptase polymerase chain reaction (RT-PCR) testing. Half of the 24 patients from whom samples were evaluable for conversion RT-PCR became disease-negative after treatment. Furthermore, 29 of 31 patients remained in complete remission at a median follow-up period of 36 months. Although that was not a randomized trial, the results suggest that lintuzumab may reduce the number of standard chemotherapy courses required to achieve long-term remission in acute promyelocytic leukemia (APL) [47] (Table 2).

**Table 2:** Monoclonal antibodies targeting myeloid leukemia.

MAb	Target Antigen	Type	Indication
Gemtuzumab	CD33	IgG4 Calicheeam immunotoxin (y1)	AML
Lintuzumab	CD33	IgG1 Humanized	Under investigation
A3D8 Anti-CD44	Adhesion glycoprotein	Murine IgG1	Under investigation
Anti-CD45 YTH2568	Leukocyte protein tyrosine phosphatase	IgG1Radio-labeled 131I murine IgG1	Under Investigation
Anti CD 66	CEA family granulocyte antigen	Radio labeled 188Re 90Y murine IgG1	Under Investigation
Anti CD 123	CD123(IL-3Ra)	Murine (pseudomonas exotoxin A immunotoxin)	Under Investigation

### 4.3. Anti-CD45 and -CD66 Antibodies

Recent findings suggest that the MAbs against the CD45 and CD66 antigens may play a role in AML treatment. Radio labeled MAbs against CD45 and CD66 have also been used to intensify conditioning regimens prior to stem cell transplantation. The most promising results were obtained with radio labeled anti-CD45 antibodies [46]. In addition, antibodies against CD66 have been used to deliver targeted radiation to hematopoietic tissues in patients with advanced myeloid malignancies. Both the unlabeled MAbs and their immunoconjugates appear to have a limited role if used as single agents to treat AML, but they hold promise as potentially useful additions to conventional therapy once the optimal dosing and timing have been defined. 

### 4.4. Anti-CD2 Antibody

*Siplizumab* is a humanized MAb recognizing human CD2 that has been studied as possible therapy for psoriasis and as prophylaxis for graft-*versus*-host disease (GVHD). In a phase I trial, 29 patients with various T cell leukemia and lymphomas were treated with antibodies; 23 of them received siplizumab on two or three consecutive days every two weeks. Flow cytometry demonstrated saturation, and down-modulation of CD2 on the surface of the peripheral blood T cells. The response to treatment included nine PRs and two CRs. All patients but one, showed a decrease in circulating CD4^+^, CD8^+^, and NK cells. Cytomegalovirus reactivation was common, but there was no disease detected. In addition, the treatment was complicated by Epstein-Barr virus infection in four patients [52].

### 4.5. Anti-CD3 Antibody

*Muromonab* is a murine IgG2A MAb directed against the CD3 subunit that has been approved for treatment of acute renal allograft rejection. It is also used to deplete T cells from bone marrow allograft to reduce of the risk of GVHD [53]. The use of muromonab leads to the clearing of T cells from the peripheral blood and lymphoid tissue, induces apoptosis, and redirects T cells in other compartments. It has also shown limited clinical activity against T cell lymphoblastic leukemia. Treatment with muromonab is associated with adverse effects that include suppression of immunity and increased risk of infection and secondary malignances [54].

### 4.6. Anti-CD4 Antibody

*Zanolimumab* is an anti-CD4 MAb that has been evaluated in patients with mycosis fungoides and Sezary syndrome. In those studies, it was found to cause lymphopenia. Thirteen of 38 patients with mycosis fungoides and two of nine patients with Sezary syndrome developed dose-dependent CD4 lymphopenia and suffered infectious complications [55].

### 4.7. Anti-CD25 Antibody

*Daclizumab:* is an anti-CD25 antibody in which approximately 90% of the murine IgG2a has been replaced with the human IgG1κ sequence. This MAb has been approved for treatment of allograft rejection in combination with other agents. Many trial of this drug in HTLV-1-associated adult T cell lymphoma found some clinical benefit. In one study, of 19 patients given murine anti-Tac, 2 achieved a CR and four had a PR. To further test the killing ability of this MAb, murine anti-Tac was chelated onto the beta-emitting radioisotope Yttrium-90, and 18 patients with ATL were treated with this radio labeled antibody; two achieved a CR and seven achieved a PR [56] (Table 3).

**Table 3:** Monoclonal antibodies for treatment of T call lymphoma leukemia.

Moab	Target Antigen	Isotype	Approval	Indications
Siplizumab	CD2	Human IgG1	Phase I-II	CD 2 T cell leukemia/ lymphoma
Muromonab-CD3	CD3	MURINE IgG2a	FDA approved	Renal, allograft rejection
Zanolimumab	CD4	Human IgG1	Phase I-III	CTCL/Sezary syndrome, T-cell lymphoma
Daclizumab	CD25	Humanized IgG2a	Phase II-III	HTLV-1-associated ATL
LMB-2	CD25	Murine anti-Tac(Fv)	Phase I-II CD25	Lymphoproliferative malignancy
Mik- β1	CD122	Humanized IgG1	Phase I	T-LGL leukemia

## 5. Conclusions

MAbs have become an integral part of the management of leukemias. Great progress has been made in this field and numerous promising MAbs are undergoing clinical development. It is likely that in the years to come novel MAbs will be precisely tailored to the individual characteristics of each patient and play a major role in the treatment of leukemias.
